# Comparison of two approaches of infraclavicular brachial plexus block for orthopaedic surgery below mid-humerus

**DOI:** 10.4103/0019-5049.65362

**Published:** 2010

**Authors:** Vikas Trehan, Uma Srivastava, Aditya Kumar, Surekha Saxena, Chandra Sekar Singh, Ankit Darolia

**Affiliations:** Department of Anaesthesia and Critical Care, SN Medical College, Agra, India

**Keywords:** Coracoid approach, infraclavicular brachial plexus block, mid-clavicular approach

## Abstract

The brachial plexus in infraclavicular region can be blocked by various approaches. Aim of this study was to compare two approaches (coracoid and clavicular) regarding success rate, discomfort during performance of block, tourniquet tolerance and complications. The study was randomised, prospective and observer blinded. Sixty adult patients of both sexes of ASA status 1 and 2 requiring orthopaedic surgery below mid-humerus were randomly assigned to receive nerve stimulator guided infraclavicular brachial plexus block either by lateral coracoid approach (group L, *n* = 30) or medial clavicular approach (group M, *n* = 30) with 25–30 ml of 0.5% bupivacaine. Sensory block in the distribution of five main nerves distal to elbow, motor block (Grade 1–4), discomfort during performance of block and tourniquet pain were recorded by a blinded observer. Clinical success of block was defined as the block sufficient to perform the surgery without any supplementation. All the five nerves distal to elbow were blocked in 77 and 67% patients in groups L and M respectively. Successful block was observed in 87 and 73% patients in groups L and M, respectively (*P* > 0.05). More patients had moderate to severe discomfort during performance of block due to positioning of limb in group M (14 vs. 8 in groups M and L). Tourniquet was well tolerated in most patients with successful block in both groups. No serious complication was observed. Both the approaches were equivalent regarding success rate, tourniquet tolerance and safety. Coracoid approach seemed better as positioning of operative limb was less painful, coracoids process was easy to locate and the technique was easy to learn and master.

## INTRODUCTION

Infraclavicular brachial plexus block (ICPB) was introduced in early 20^th^ century as an alternative to axillary and supraclavicular approaches. However, this approach was not utilised despite its advantages of less complications and more consistent block until Raj *et al*. introduced this in 1973.[1] But Raj’s technique could also not gain widespread use probably due to unreliable results[2] and lack of precision in needle placement.[3] Since then several variations on the technique of ICPB have been described with various surface landmarks, site of needle insertion and recommendations for needle direction.[4–9] The two most common approaches are medial approach around the middle of clavicle and lateral approach around the coracoid process. Others include parasagittal and pericoracoid approaches. Out of all the approaches, coracoid approach is most popular.[10] It has gained popularity because of presence of a consistent bony land mark, less chances of vascular puncture or pneumothorax and adequate neural blockade.[5]

The aim of our study was to compare two approaches of ICPB (lateral coracoid and medial clavicular) for orthopaedic surgery of elbow, forearm, wrist or hand. The outcomes studied were clinical success rate of block, discomfort during performance of block, complications and pain related to tourniquet.

## METHODS

After institutional approval and informed written consent, 60 adult patients aged between 18 and 60 years of both sexes requiring surgery below mid-humerus were selected for this prospective, randomised, observer blinded study. This study was done from 1 October 2008 to 2 September 2009. Patients with conditions precluding regional block (local infection, coagulopathy) were excluded. The patients belonging to ASA status I and II and who could cooperate were randomly divided into two equal groups. Group allocation was done using the last digit of their medical sheet. The patients with an odd numbers received ICPB using lateral coracoid approach of Wilson *et al*,[5] (group L) and patients with even numbers received block by medial clavicular approach described by Borgeat *et al*[7] (group M). All the blocks were performed in a room adjacent to the main OR with all routine monitoring. Each patient received 0.25 mg oral alprazolam, about 2–3 hours before block. An IV line was secured and 50–100 *µ*g of fentanyl was given according to patient’s age and weight. The block was performed with the patients in supine position with the head turned to the opposite side of the limb to be operated.

In group L, the operative limb was laid in neutral position along the body. After sterile preparation, the coracoid process was identified by palpation and a point 2 cm medial and 2 cm caudal to coracoid process was marked and 1–2 ml of 1% lignocaine was infiltrated. Insulated needle was inserted perpendicular to the skin. In group M, with the patient lying supine and arms in neutral position along the body, a point bisecting a line joining the ventral acromial process of scapula and jugular notch was marked. The point of emergence of axillary artery in fossa axilaris was identified next. To perform the block, the arm was abducted to 90° and elevated to approximately 30° using a pillow. A point was marked 1 cm below inferior border of clavicle at its midpoint and infiltrated with lignocaine. The insulated needle was inserted directed laterally angled 45°–60° to the skin towards emergence of axillary artery in axilla as close as possible to the lateral border of pectoralis major muscle.

In both the groups, the block was performed using a nerve stimulator (Stimuplex, B. Braun Melsungen, Germany) connected to the proximal end of 50-mm, 22-G insulated needle. The needle was advanced until a muscle distal to deltoid was stimulated. The initial stimulating current was set at 2.0 mA and it was gradually reduced and the needle was slowly inserted till the current of 0.05 mA still elicited a slight distal motor response. A response was considered proximal if contraction of the triceps, biceps, flexor carpi radialis or flexor carpi ulnaris was elicited and distal if flexion or extension of wrist or fingers was elicited. In each patient, distal response was desired, but if it could not be obtained, proximal response was taken as satisfactory. Then 25–30 ml of 0.5% bupivacaine was injected slowly with intermittent aspiration. The patients were transferred to OR after 30 minutes and any block that was inadequate for surgery were supplemented with local infiltration at the surgical site, IV fentanyl or GA, if required.

Sensations in the distribution of ulnar, radial, median, musculocutaneous and medial cutaneous nerve of arm were evaluated 20 minutes after block using 26-G needle. Motor block was assessed 30 minutes after block and graded as follows: grade 1 = ability to flex and extend the forearm, grade 2 = ability to flex or extend only wrist and fingers, grade 3 = ability to flex or extend only fingers and grade 4 = inability to move forearm wrist or fingers. Successful block was defined when the surgery was possible without any additional supplementation. After the block was performed, the patients were enquired about discomfort during the block procedure (due to positioning or needle insertion) as none, mild, moderate or severe. Other observations studied were depth of the needle inserted (distance between puncture site and drug injection), tourniquet pain, duration of surgery and complications of block (intravascular injection, arterial puncture, pneumothorax, Horner’s syndrome, etc). The patients were monitored throughout the surgery, after tourniquet deflation and postoperatively by continuous pulse oximetry, ECG, heart rate and non-invasive blood pressure very 15 minutes.

### Statistical analysis

Sample size calculation was based on a projected difference of 20% in success rate among the two groups. Based on this, we calculated a sample size of minimum 30 patients per group, which would permit a type 1 error of alpha = 0.05 with a type II error of beta = 0.5 and power of 0.8. Data were presented as mean and SD or percent of patients. Continuous variables were analysed using two sample *t*-test while the chi-square test was used to compare categorical variables. Statistical difference was defined as *P* < 0.05.

## RESULTS

The demographic and other data are depicted in Table 1. The patients matched regarding demographic data, duration and region of surgery in the two groups.

**Table 1:** Demographic and other data (mean ± SD)

	Group L (*n* = 30)	Group M (*n* = 30)	*P* value
Age (years)	33 ± 10.02	36 ± 13.08	0.9459
M/F	26/4	26/4	
Weight (kg)	64 ± 3.97	62 ± 4.60	0.2976
Stimulating current (mA)	0.47 ± 0.062	0.46 ± 0.060	0.7353
Surgical duration (minutes)	43 ± 14.53	40.3 ± 13.3	0.956
Needle depth (cm)	3.14 ± 0.242	3.63 ± 0.195	0.001
Surgical region			
Hand and wrist	8 (27)	7 (23)	
Forearm	17 (57)	16 (53)	
Elbow	5 (17)	7 (23)	

Figures in parentheses are in percentage

Success rate defined as operability without any supplementation was 87% in coracoid approach (group L) and 73% in clavicular approach (group M), but difference was not significant statistically (*P* > 0.05). In the patients with block failure, two and five patients required surgical infiltration by the surgeon for surgery to be accomplished and two and three patients required GA in groups L and M, respectively [Table 2]. Tourniquet was applied in 22 (group M) and 23 (group L) patients. No tourniquet pain was reported in 20 (group L) and 19 patients (group M) with successful block [Table 2].

**Table 2:** Study results

	Group L	Group M	*P* value
Block sufficient for surgery	26 (87)	22 (73)	0.19
Local infiltration and/IV fentanyl	2	5	
GA	2	3	
Tourniquet applied (*n*)	22	23	
No sensation	17	14	0.075
Sensation, no pain	3	5	0.12
Pain	2	4	0.35
All five nerves distal to elbow blocked	23 (77)	20 (67)	0.523
Degree of motor block (grades 1,2,3,4)	2, 1, 5, 22	3, 4, 8, 15	0.26

Figures in parentheses are in percentage

In group L, majority of the patients had no discomfort or only mild discomfort during performance of block (22 vs. 16) while more number of patients had moderate or severe discomfort during performance of block in group M compared to group L (14 vs. 8) though the difference was not significant (*P* = 0.337) [Table 3]. The discomfort was mainly due positioning of limb. The degree of motor block was similar in both the groups (*P* = 0.26) [Table 4]. The incidence of block-related complications was very low in both the groups. No patients had pneumothorax or arterial puncture in any group. One patient in each group had venous puncture. All the patients remained haemodynamically stable during the operation and postoperatively.

**Table 3:** Discomfort during performance of block

	Group L	Group M	*P* value
Nil	10 (33.3)	5 (16.7)	0.13
Mild	12 (40)	11 (36.67)	0.79
Moderate	6 (20)	10 (33.3)	0.24
Severe	2 (6.7)	4 (13.33)	0.39

**Degree** of freedom = 3, *P* value = 0.337, χ^2^ = 3.377, Figures in parentheses are in percentage

**Table 4:** Degree of motor block

Degree of motor block	Group L	Group M	*P* value
Grade 1: Flex and extend forearm	2 (6.7)	3 (10)	0.64
Grade 2: Flex and extend only wrist and fingers	1 (3.33)	4 (13.33)	0.16
Grade 3: Flex and extend only fingers	5 (16.67)	8 (26.67)	0.35
Grade 4: No movement of forearm, wrist or fingers	22 (73.33)	15 (50)	0.06

DF = 3, *P* value = 0.26, χ^2^ = 4.017, Figures in parentheses are in percentage

## DISCUSSION

Results of this study indicate that successful block was obtained in more number of patients when ICPB was performed using coracoid approach (group L) than in patients in whom the block was performed using midclavicular approach (group M), although the difference did not reach statistical significance. The incidence of complications was very low in both the groups and tourniquet tolerance was similar in both of them. Patient’s discomfort during performance of block was higher in group M as the operative limb was moved during positioning for the block.

The reported success rate of ICPB varies greatly following coracoids and other approaches, ranging between 44 and 100%.[2,6–8,11,12] This could be due to variation in type of motor response accepted (proximal or distal) for local anaesthetic injection, single or multiple neuro-stimulation, definition of block success, area of surgery (forearm, wrist or hand), etc.

We could not obtain distal motor response (flexion of wrist or fingers) in each case, which is an important predictor for better results irrespective of the approach.[2,6–9,11,13] Borgeat *et al*. (2001) reported a success rate of 44% when proximal response was accepted for local anaesthetic injection, compared to 97% when distal motor response was accepted. Desroches (2003) reported 13 unsuccessful blocks from 75 patients with proximal stimulation. But in fact, in clinical practice it is not uncommon to obtain a proximal response[14,15] and repeated blind attempts to seek distal muscle response may result in vascular puncture, pneumothorax and discomfort to patients.[14] There is no clear anatomic basis in literature to explain the notion that distal stimulation is associated with better success.[7,13] The suggested causes may be following. Proximal response of biceps can be due to stimulation of musculocutaneous nerve, which often leaves the lateral cord at or above the level of infraclavicular region.[2,8] Secondly, when distal response is obtained, the needle is more centrally placed, resulting in even diffusion of local anaesthetic.[2,7,14,16] Some authors emphasise that double or multiple stimulation[11,17,18] improves success rate of ICPB, whereas others suggest that stimulation of median nerve,[13] posterior cord[16] or all three cords[11] causes less chances of failure.

Besides this, the definition of successful block varies. Some authors define the success of block as complete sensory and motor block of all the five nerves below the elbow[2,7–9,11] while others define success rate as performing the surgical procedure without any supplementation.[16,19] In this study, the block sufficient to allow surgery without any supplementation was considered as successful. The operation site could also influence the probability of block success, for example, hand surgery can be performed successfully with blockade of two to three nerves,[11] but blockade of all nerves is required for surgery in the forearm or elbow. A recent study[20] has shown that failure of block could be due to inadequate spread of local anaesthetic because of the presence of septa in neurovascular sheath in infraclavicular region.

Tourniquet was well tolerated by most patients without any additional infiltration with successful block, suggesting proximal extension of blockade of medial cutaneous nerve of arm. Jandard *et al*,[8] found a correlation between sparing of median cutaneous nerve of arm and tourniquet pain and advised to block this nerve separately. But we did not require this in any patient, an observation in agreement with the previous study.[2]

The incidence of block-related complications was very low in our patients by either approach. Only three of our patients had vascular puncture. Our results are similar to that seen in many previous reports.[2,6,7] None of the patients had pneumothorax. This was probably due to more lateral approach in group L and angulated direction of needle in group M. Our results are in agreement with other studies.[5,7,8,17,18]

Both the approaches provided more or less equivalent success rate with infrequent incidence of complications. But the disadvantage of the clavicular technique was that the positioning of the limb was painful in patients who had fracture in forearm resulting in discomfort during the block. Also, the palpation of anterior achromial process in some patients was difficult. The advantages of coracoid approach were that no movement of limb was required and coracoid process was easily identified in all patients. Besides, as the direction of the needle was perpendicular to skin, the technique was easy to learn. Although it is generally agreed that infraclavicular block is safe regarding pulmonary complications, occasional case reports of pneumothorax have been published.[21,22] A thorough understanding of relevant anatomy and anatomic variability is necessary to avoid this rare complication in a relatively safe technique of infraclavicular plexus block. Ultrasound can be a significant aid in increasing the success rate and reducing the complications.[12,15]
